# Permeability across a novel microfluidic blood-tumor barrier model

**DOI:** 10.1186/s12987-017-0050-9

**Published:** 2017-01-23

**Authors:** Tori B. Terrell-Hall, Amanda G. Ammer, Jessica I. G. Griffith, Paul R. Lockman

**Affiliations:** 1 Department of Basic Pharmaceutical Sciences, School of Pharmacy, West Virginia University HSC, 1 Medical Center Dr., Morgantown, WV 26506 USA; 2 WVU Cancer Institute Research Laboratories, West Virginia University HSC, Morgantown, WV 26506 USA

**Keywords:** Efflux, p-glycoprotein, Permeability, Metastasis

## Abstract

**Background:**

The lack of translatable in vitro blood-tumor barrier (BTB) models creates challenges in the development of drugs to treat tumors of the CNS and our understanding of how the vascular changes at the BBB in the presence of a tumor.

**Methods:**

In this study, we characterize a novel microfluidic model of the BTB (and BBB model as a reference) that incorporates flow and induces shear stress on endothelial cells. Cell lines utilized include human umbilical vein endothelial cells co-cultured with CTX-TNA2 rat astrocytes (BBB) or Met-1 metastatic murine breast cancer cells (BTB). Cells were capable of communicating across microfluidic compartments via a porous interface. We characterized the device by comparing permeability of three passive permeability markers and one marker subject to efflux.

**Results:**

The permeability of Sulforhodamine 101 was significantly (p < 0.05) higher in the BTB model (13.1 ± 1.3 × 10^−3^, n = 4) than the BBB model (2.5 ± 0.3 × 10^−3^, n = 6). Similar permeability increases were observed in the BTB model for molecules ranging from 600 Da to 60 kDa. The function of P-gp was intact in both models and consistent with recent published in vivo data. Specifically, the rate of permeability of Rhodamine 123 across the BBB model (0.6 ± 0.1 × 10^−3^, n = 4), increased 14-fold in the presence of the P-gp inhibitor verapamil (14.7 ± 7.5 × 10^−3^, n = 3) and eightfold with the addition of Cyclosporine A (8.8 ± 1.8 × 10^−3^, n = 3). Similar values were noted in the BTB model.

**Conclusions:**

The dynamic microfluidic in vitro BTB model is a novel commercially available model that incorporates shear stress, and has permeability and efflux properties that are similar to in vivo data.

## Background

The occurrence of brain metastases in breast cancer patients is approximately 10–16% [1]. Due to improvements in chemotherapy, the overall survival of breast cancer patients has increased. Unfortunately with prolonged survival the incidence of patients developing symptomatic brain metastases has also increased. One of the leading complications of brain metastases is the inability of drugs to reach the tumor at concentrations adequate to induce cytotoxicity. This is due, in part, to the presence of a partially intact blood–brain barrier (BBB).

The BBB is a complex anatomical network, functioning to strictly regulate the movement of molecules and ions from the blood to the brain and back. In addition, the BBB serves as the conduit to supply the brain with the essential nutrients it needs, while facilitating the excretion of waste products through efflux [2]. The hallmark of the BBB is the presence of endothelial cells that are tightly connected by tight junction protein complexes, which are composed of claudins, occludins, and junction adhesion molecules [3]. In addition to endothelia, the BBB has a thick basal membrane with pericytes and astrocytic foot processes in close proximity [4, 5]. The net effect of this anatomical structure results in the transendothelial electrical resistance (TEER) of brain capillaries being ~2000 O*cm^2^, in comparison to 2–20 O*cm^2^ in peripheral capillaries [6, 7]. In addition to the structural components, the BBB is highly enriched in efflux transporters that actively restrict the entry a large and diverse set of lipophilic solutes from accumulating in the brain [8, 9].

When metastatic cancer cells invade the CNS, they may eventually colonize and proliferate into a larger tumor mass. Once the lesion has grown to a point that it has areas of hypoxia, the tumor will secrete high amounts of vascular endothelial growth factor in an attempt to develop a new blood supply [10, 11]. This vasculature (blood-tumor barrier; BTB) is different than the BBB predominantly because astrocytes, pericytes, and neurons are no longer in close proximity to the capillary. It is hypothesized that these anatomical changes result in vasculature that has greater permeability than the BBB [12]. The BTB may also have a somewhat different and varied expression of efflux transporters depending on the CNS malignancy [13–15]. Despite the apparent breakdown of the BBB in the presence of a tumor [16], the BTB still limits drug movement into the CNS lesion significantly more than in peripheral tumors.

Currently, there are no widely validated in vitro models of the BTB. The most widely used in vitro BBB model, which also has been used to model the BTB, is a transwell insert system. Briefly, the model consists of an upper chamber with endothelial cells grown on the surface separated from a lower chamber that may or may not contain astrocytes and or cancer cells. The two chambers are separated by a porous membrane [17, 18]. Drug movement is modeled by measuring accumulation in the lower chamber versus time.

However, the transwell model has limitations. First, there is a lack of flow exerted on the endothelia resulting in poor cell morphology and a “leakier” barrier compared to in vivo data [17–21]. Second, endothelial cells do not uniformly attach to the outer walls of the insert, leaving gaps between endothelial cells and the edge of the insert, also resulting in increased permeability [22]. Third, an unstirred water later forms on the surface of the endothelia, which results in increased permeability for hydrophilic drugs and decreased permeability for lipid soluble drugs [23, 24].

Herein we characterize novel in vitro microfluidic model of the BTB and BBB (as a reference point) using a co-culture of human umbilical endothelial cells (one of a number of endothelial cell lines used in a number of BBB studies [25, 26]) and brain metastases cells. This model incorporates flow during culture of the endothelia and has a micro-tubular lumen, which in other work has substantially reduced limitations seen in transwells [27–31]. This model is unique from other flow based models in that it allows for a co-culture or triple culture of relevant cells, it is easily duplicated, it is commercially available and provides a cost-effective solution for running multiple and parallel assays.

## Methods

### Microfluidic device

Co-culture idealized microvascular networks used in this study were obtained from SynVivo Inc. Huntsville, AL. The device consists of a central compartment (basolateral) that is comprised of the brain tissue cells (astrocytes, pericytes, neurons) and the outer compartment (apical) that is comprised of the endothelial cells and provides perfusion similar to physiological fluid flow conditions. The outer compartments and central compartment are separated by an interface with a series of 3 μm pores along the length, replacing the use of membranes in conventional models.

### Chemicals

Sulforhodamine 101 acid chloride (Free TRD), Rhodamine 123 (Rho123), Texas Red 3000 MW Dextran (TRD 3 kDa), and Texas Red 70,000 MW Dextran (TRD 70 kDa) were purchased from ThermoFisher Scientific (Grand Island, NY). Verapamil was purchased from Sigma (St. Louis, MO). Cyclosporine A was purchased from Toronto Research Chemicals Inc. (Toronto, Canada). All other chemicals used were of analytical grade and were used as supplied.

### Cell culture

Human umbilical vein endothelial cells (HUVECs) were purchased from Lonza (Allendale, NJ). CTX-TNA2 rat brain astrocytes were kindly donated by Dr. Jim Simpkins (West Virginia University, Morgantown, WV). Met-1 murine metastatic breast cancer cells were a kind gift from Dr. Alexander Borowsky (UC Davis, Sacramento, CA). All cells were maintained in endothelial basal medium-2 (EBM-2) supplemented with the EGM-2 BulletKit from Lonza (Allendale, NJ). Cells were grown in a 37 °C humidified incubator with 5% CO_2_ until ~85% confluent.

### Cell culture in microfluidic chip

Matrigel (40 μg cm^−2^, EMD Milipore, Billerica MA) was injected into the central compartment and allowed to sit covered in ice for approximately 1 h, after which serum-free media was promptly injected to wash the central compartment. Fibronectin (200 μg mL^−1^, EMD Milipore, Billerica MA) was then injected into one of the outer sides of the device and allowed to incubate at 37 °C overnight. Prior to the seeding of all cells, the device was flushed with EBM-2 media. Astrocytes/Met-1 cells were harvested using TrypLE Select (ThermoFisher, Waltham MA) and re-suspended into a final concentration of ~1 × 10^7^ mL^−1^ cells for injection, and were seeded at a flow rate of 10 μL min^−1^ in the central compartment using a Pump 11 Elite Nanomite programmable syringe pump (Harvard Apparatus, Holliston MA). The inlet port tubing was clamped when cells reached a central compartment density of ~50%, and chip was transferred to a CO_2_ incubator at 37 °C to allow cells to attach for 2 h. HUVECs were harvested using TrypLE Select (ThermoFisher, Waltham MA) in the same process described above, re-suspended to a concentration of ~1 × 10^7^ mL^−1^, and seeded into the outer compartment previously coated with Fibronectin at a flow rate of 6 μL min^−1^ using a Pump 11 Elite Nanomite programmable syringe pump (Harvard Apparatus, Holliston MA). Inlet port tubing was clamped when HUVECs reached an intra-outer compartment density of ~90%, then chip was transferred to a CO_2_ incubator at 37 °C and cells were allowed to attach for 24 h, with the exception of media refreshment. After 6 h of incubation, medium in central and both outer compartments was replaced with fresh EBM-2 medium. Media replacement was repeated again at 24 h. Astrocyte/Met-1 cells were maintained in the central compartment under static conditions in EBM-2 medium while EBM-2 medium was prepared in syringes mounted on a programmable PHD 2000 syringe pump (Harvard Apparatus, Holliston MA) and then connected to the chips through ~12 in. of sterile Tygon tubing (Harvard Apparatus, Holliston MA). This medium was flowed at a flow rate of 0.02 μL min^−1^ over the seeded HUVECs in the outer compartment for 4 h, then increased to 0.05 μL min^−1^ after 4 h, and finally to 0.1 μL min^−1^ after 4 more hours, equivalent to 1.9 × 10^−3^ dynes cm^−2^ [32] This flow rate of 0.1 μL min^−1^ was then maintained for 24 h.

### In vitro transport studies

EBM-2 Medium was incubated in a BD Luer-Lok Syringe with either Free TRD (600 mg mL^−1^), TRD 3 kDa (600 mg mL^−1^), TRD 70 kDa (600 mg mL^−1^), or with R123 (600 mg mL^−1^) in the presence or absence of known P-gp inhibitors (verapamil: 50 mM, Cyclosporine A: 10 mM) and mounted on a programmable PHD 2000 syringe pump (Harvard Apparatus, Holliston MA), with syringes connected to chips through sterile Tygon tubing (Harvard Apparatus, Holliston MA). Permeability was measured through the injection of desired tracer into the outer compartment at 0.1 μL min^−1^ for a total of 90 min while brightfield images (acquired at a 25 ms exposure) and fluorescent images (acquired at a 200 ms exposure) were acquired every 2 min. Permeability of each tracer was determined using NIS Elements Imaging Software. Using linear regression (Prism 6.0), the slope of the best-fit line was used to represent the relative k_in_, or rate of accumulation, of fluorescence in the central compartment (comparable to the concentration of drug found in normal brain) divided by the accumulation of fluorescence in the outer compartment (comparable to the concentration of drug found in the plasma of the BBB vasculature). Unless otherwise stated, data are presented as mean ± SEM.

### Quantification of fluorescent tracers using fluorescent microscopy

Chips were mounted on an automated stage enclosure, maintained at 37 °C with 5% CO_2_, on a Nikon Eclipse TE2000-E Live Cell Sweptfield Confocal microscope (Melville, NY). Acquisition of images and fluorescence was achieved through the utilization of a Photometrics CoolSnap HQ2 Monochrome CCD Camera (Tucson, AZ) with a 20×/0.75 Plan Fluor Phase Contrast objective with a total field of 6 × 8, stitching images using brightfield with a 10% overlay. Brightfield and fluorescent images were taken every 2 min for 90 min. Excitation and emission of Free Texas Red, Texas Red 3 and 70 kDa, was obtained using the TRITC epiflourescence filter (peak fluorophore excitation is 596 nm and emission is 615 nm); excitation filter wheel of 555/25×, emission filter wheel of 605/52 m and dichromatic mirror at 89,000 sedat quad. The excitation and emission of Rhodamine 123 (±Cyclosporine A or Verapamil) was obtained using the FITC epiflourescence filter (peak fluorophore excitation is 511 nm and emission is 534 nm); excitation filter wheel of 490/20×, emission filter wheel of 525/36 m and dichromatic mirror at 89,000 sedat quad.

### Kinetic analysis

Unidirectional uptake transfer constants (k_in_) were calculated from the following relationship to the linear portion of the uptake curve:

1 $$\left( {{\text{C}}_{\text{CC}} + {\text{ C}}_{\text{PF}} } \right) \, /{\text{ C}}_{\text{PF}} = {\text{ k}}_{\text{in}} \left( {\text{t}} \right) \, + {\text{ O}}_{\text{C}}$$

where C_CC_ is the sum intensity of fluorophore in the region of interest in the central compartment (au) at the end of perfusion, C_PF_ is the sum intensity of fluorophore (au) in the region of interest within the outer compartment, t is the perfusion time in minutes, and O_C_ is the extrapolated intercept [T = 0 min; “outer compartment volume” (au)]. After the determination of a perfusion time where an adequate amount of fluorescent marker was allowed to pass into brain, while still remaining in the linear uptake zone, k_in_ was determined [33].

### Statistical analysis

The slope of the line (k_in_) was determined with linear regression using best-fit values. One-way ANOVA analysis and unpaired t test with Welch’s correction, followed by an F test to compare variances were used for the comparison of the k_in_ values between unrestricted diffusion, BBB, and BTB among each tracer and with Rho123 in absence and presence of inhibitors. For all data, errors are reported as standard error of the mean unless otherwise indicated. Differences were considered statistically significant at the p < 0.05 levels (GraphPad Prism version 6.00 for Mac, GraphPad Software, San Diego, CA, USA).

## Results

**Fig. 1:**
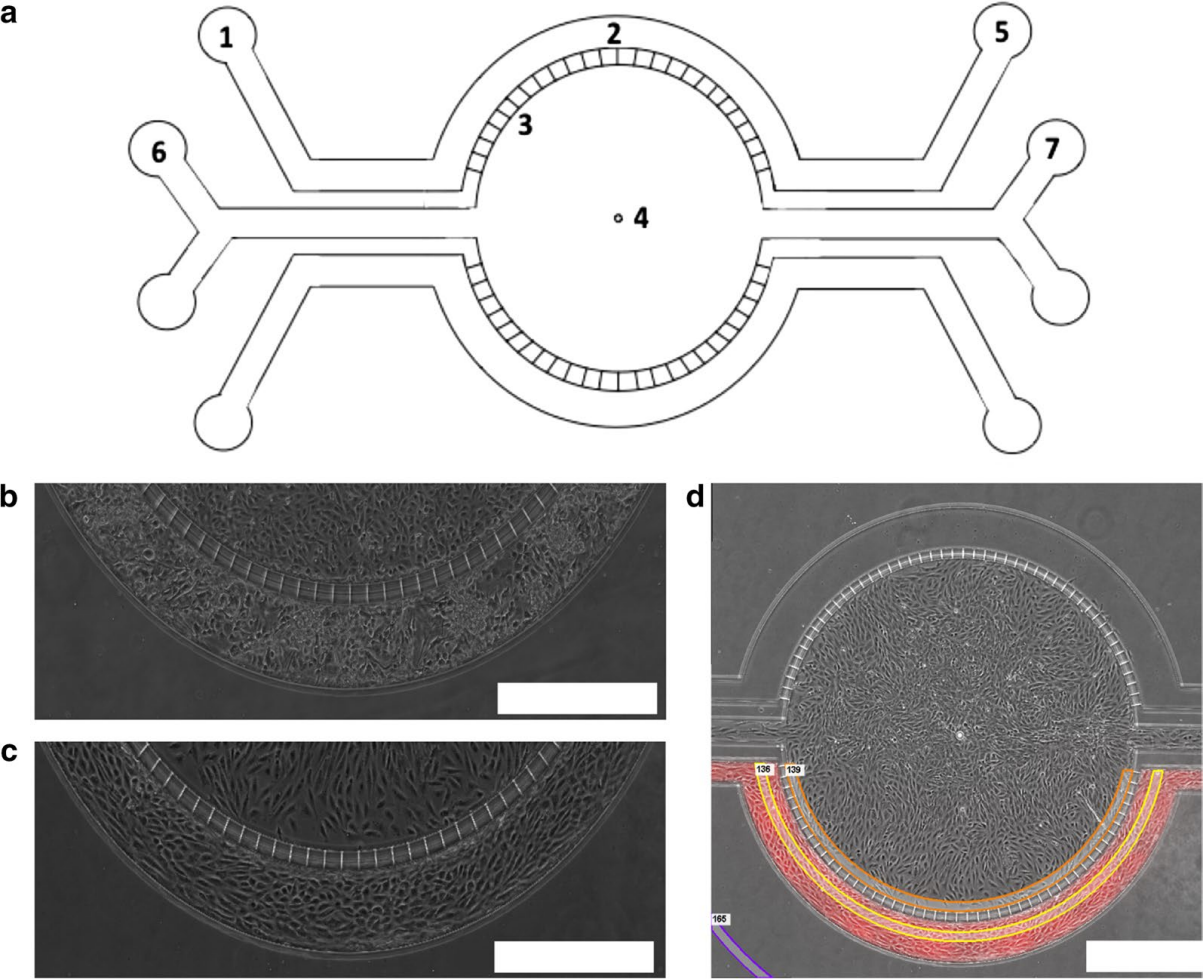
**a** Schematic of SynVivo BBB microfluidic chip: (*1*) inlet port where media with or without tracer is flowed through the outer compartment to change media for HUVECs. (*2*) Outer compartment, containing HUVECs. (*3*) 3 μm pores, to allow diffusion of media and tracer between the central and outer compartments. (*4*) Central compartment, containing astrocytes or cancer cells. (*5*) Outlet port where perfusate from the outer compartment is collected. (*6*) Inlet port for central compartment, used to seed and change media for the astrocytes/cancer cells in the central compartment. (*7*) Output ports where perfusate from the central compartment is collected. **b** Morphology of astrocytes in the central compartment and HUVECs in the outer compartment without the addition of flow (**c**) morphology of astrocytes in the central compartment and HUVECs in the outer compartment with the addition of flow. **d** Representation of where the regions of interest (ROI) measurements are taken for data analysis. *White rectangle scale bars* 500 μm

**Fig. 2:**
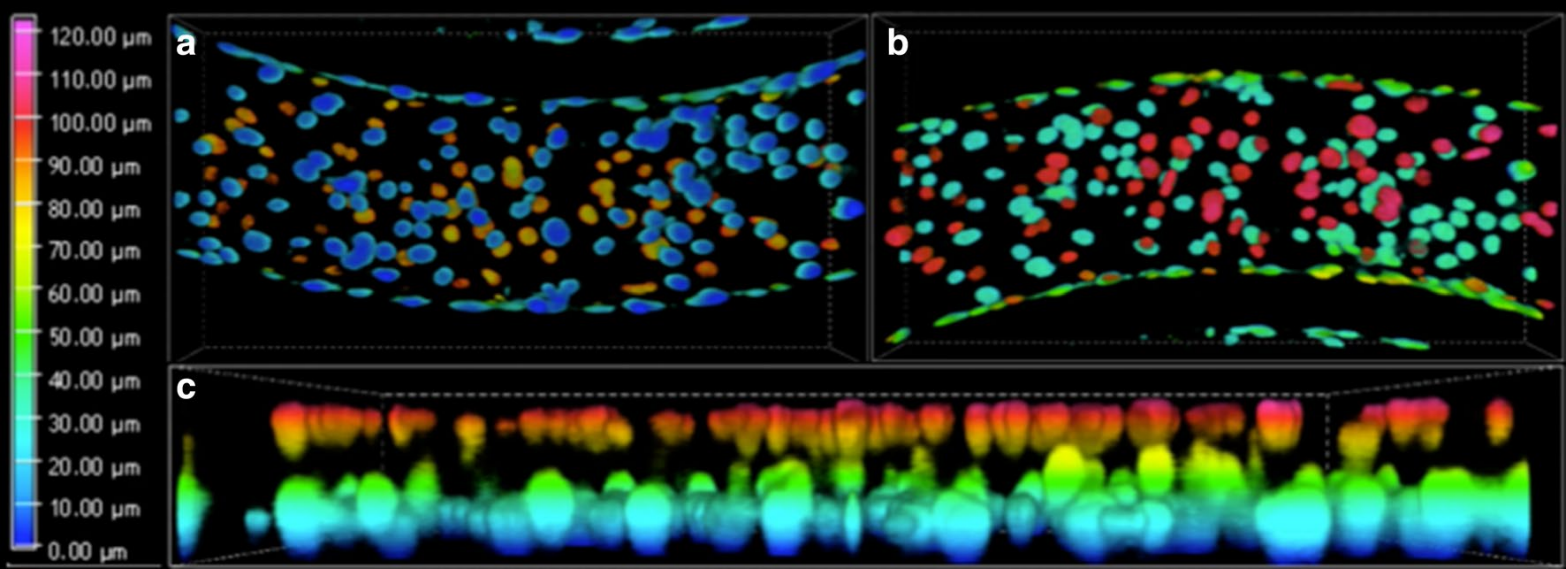
3-dimensional confocal images of DAPI labeled HUVECs in the outer compartment demonstrating a 360^o^ coating of cells. The nuclei of the HUVECs are seen on the *bottom* (**a**) and *top* (**b**) and in a *side view* (**c**)

In this study, we evaluate transfer rates of Free TRD, Texas Red 3 kDa, Texas Red 70 kDa, and Rho123 (with and without inhibitors) (Fig. 1) in a novel microfluidic BBB and BTB model as validation to previously published literature [34]. Briefly in this model, endothelial cells are seeded in the outer compartments, while astrocytes (BBB) or brain seeding breast cancer cells (BTB) are seeded in the central compartment. The porous architecture between the two compartments allows for cellular crosstalk and biochemical exchanges, while shear stress from perfusate flow facilitates development of endothelial morphology [19]. Confocal brightfield images show the differences in morphology between endothelial cells with and without flow (Fig. 1b, c). In order to verify a confluent 360° coating of endothelial cells within the outer compartment, we used a Nikon A1R Confocal on Eclipse TiE Microscope to acquire a 3D z-stack of the outer compartment. Utilizing this system, DAPI stained endothelial cells were imaged from the bottom (Fig. 2a), through to the top (Fig. 2b) showing HUVECs wrapping around the sides of the outer compartment (Fig. 2c) connecting the HUVECs on the top to the HUVECs on the bottom, verifying confluent formation of a tubular in vitro microvasculature.

**Fig. 3:**
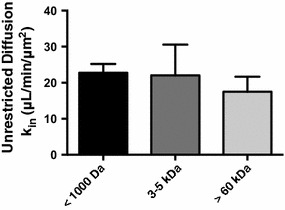
The diffusion rates of free MW tracers <1000 Da, 3–5 kDa and >60 kDa in an unrestricted, cell free microfluidic chips are shown. Statistical significance was determined using one-way ANOVA followed by Tukey’s multiple comparison tests, and student’s *t* test; n = 3–6 chips. All data represent mean ± SEM

In initial kinetic experiments, we determined unrestricted diffusion rates of difference sized molecules by perfusing solutes through microfluidic chips without endothelial cells or astrocytes/cancer cells. To quantify tracer accumulation, regions of interest were selected to determine sum fluorescence intensity in the outer compartment (ROI 136), central compartment (ROI 139), and background (ROI 165) over time (1D). ROI 165 was taken to ensure data received in the outer and central compartments were significant when compared to the background sum fluorescence. We observed (Fig. 3) that small tracers (<1000 Da) had a diffusion rate of 22.8 ± 2.5 × 10^−3^, n = 6, which was not significantly different compared to tracers of molecular weights between 3 and 5 kDa (22.1 ± 8.5 × 10^−3^, n = 3) and >60 kDa (17.5 ± 4.2 × 10^−3^, n = 3).

**Fig. 4:**
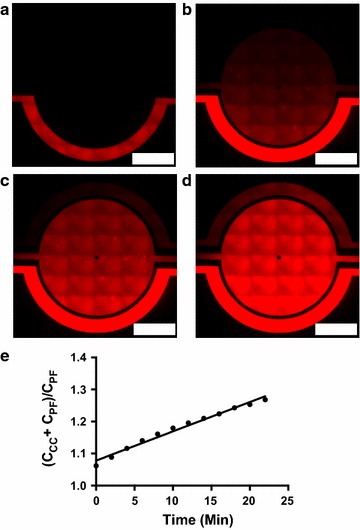
Representative timelapse images showing passive diffusion of Free TRD from the outer to the central compartment. Intensity of fluorescence increases linearly over time 0 min (**a**), 30 min (**b**), 60 min (**c**), and 90 min (**d**). **e** Linear concentration of tracer movement versus time to determine diffusion constants (K_in_). *White rectangle scale bars* 500 μm

**Fig. 5:**
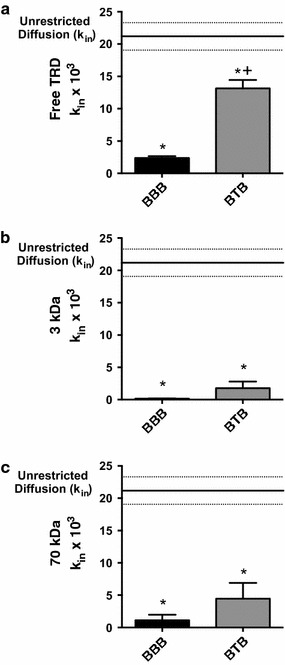
Linear central compartment accumulation of Free Texas Red (**a**), Texas Red 3 kDa (**b**), and Texas Red 70 kDa (**c**) in BBB and BTB SynVivo chip models. Images show rate of each tracer within each model. Statistical significance was determined using one-way ANOVA followed by Tukey’s multiple comparison tests, and student’s *t* test; *p < 0.05 significance between tracer and unrestricted diffusion k_in_, n = 3–6; ^+^p < 0.05 significance between BBB and BTB models, n = 3–6. All data represent mean ± SEM

In our next experiments, we qualitatively imaged Texas Red accumulation from 0 to 90 min in the BBB model (Fig. 4a–d). Linear accumulation of the dye in the central chamber of the BBB model is quantitatively shown in Fig. 4e. We then determined k_in_ values for each tracer in both the BBB and BTB model, given in units of (µL min^−1^) according to the equation found in our methods. Free Texas Red k_in_ values (Fig. 5a) for the BBB (2.5 ± 0.3 × 10^−3^, n = 6) and BTB (13.1 ± 1.3 × 10^−3^, n = 4) were significantly different (p < 0.05) between each other. Texas Red 3 kDa values (Fig. 5b) for the BBB (0.1 ± 0.1 × 10^−3^, n = 3) and BTB (1.8 ± 1.0 × 10^−3^, n = 3) and Texas Red 70 kDa values (Fig. 5c) for the BBB (1.1 ± 0.9 × 10^−3^, n = 3) and BTB (4.5 ± 2.4 × 10^−3^, n = 3) were also significant (p < 0.05) when compared to the unrestricted diffusion k_in_, but significance was not observed between the BBB and BTB models of these dyes.

**Fig. 6:**
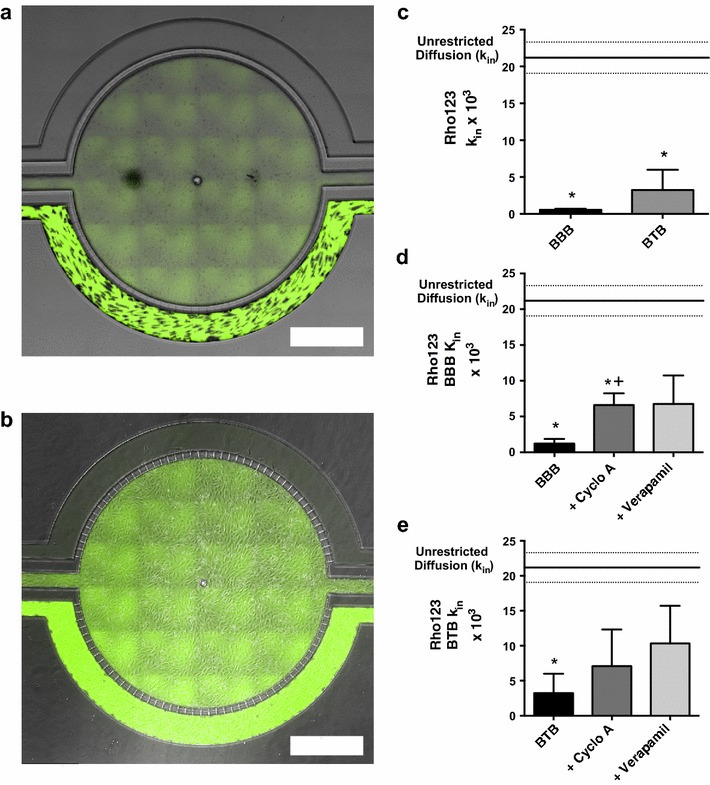
Representative brightfield image of Rhodamine 123 dye accumulation in the central compartment after 90 min of perfusion in the BBB model without an inhibitor (**a**) and with an inhibitor (**b**). Rate of fluorescent dye accumulation of Rho123 into central compartment after 90 min of dye perfusion in BBB, and BTB chips (**c**). Rate of fluorescent dye accumulation in BBB (**d**) and BTB (**e**) chips perfused with Rho123 ± P-gp inhibitors (Cyclosporine A or Verapamil). Statistical significance was determined using one-way ANOVA followed by Tukey’s multiple comparison tests, and student’s *t* test; *p < 0.05 significance between tracer and unrestricted diffusion k_in_, n = 3–4; ^+^p < 0.05 significance between BBB/BTB models and the addition of inhibitor, n = 3–6. All data represent mean ± SEM. *White rectangle scale bars* 500 μm

To determine if P-gp inhibitors alter the accumulation of P-gp sensitive fluorescent dye accumulation into the central compartment we perfused Rho123 in the absence and presence of P-gp inhibitors Cyclosporine A (10 mM), and Verapamil (50 mM)—concentrations that ensured maximal inhibition [34]. We qualitatively observed an increase in dye accumulation in the central compartment over the course of 90 min in both the BBB (Fig. 6a) and BTB (Fig. 6b) models (Fig. 6c). Quantitatively, we observed a 14-fold increase of Rho123 in the central compartment, in the presence of P-gp inhibitor Verapamil (14.7 ± 7.5 × 10^−3^, n = 3), and a significant (p < 0.05) eight fold increase of Rho123 with Cyclosporine A (8.8 ± 1.8 × 10^−3^, n = 3) when compared to control Rho123 (0.6 ± 0.1 × 10^−3^, n = 4) in the BBB model (Fig. 6d). Similarly in the BTB model, a threefold increase was observed in Rhodamine 123 permeability in the presence of P-gp inhibitor Verapamil (10.3 ± 3.1 × 10^−3^, n = 3), and a twofold increase with Cyclosporine A (7.1 ± 5.2 × 10^−3^, n = 3) when compared to Rho123 control (3.2 ± 2.8 × 10^−3^, n = 3) (Fig. 6e).

## Discussion

The results of the studies presented herein suggest that a novel microfluidic chip in part mimics the in vivo BTB with regard to passive permeability and efflux [16]. Importantly, this study demonstrates that perfusion flow through the luminal compartment improves endothelial function. This model also has potential to be used in screening assays for drug discovery and development for central nervous system disease.

Predominant in vitro BBB models have some key similarities. First, there is a presence of some type of “barrier” cell in a luminal or outer compartment (representing the vascular lumen). These cells range from primary or immortalized brain endothelial cells (most commonly rat, mouse, or human), peripheral endothelial (HUVECs), or stem-cell derived cells. In this study HUVECs were chosen as they are commonly used by a number of labs, and using a co-culture of astrocytes or even astrocyte-conditioned media alone has been shown to induce BBB-like expression of tight junctions and barrier tightness in a variety of endothelial cells [20, 29, 35]. These barrier cells typically express tight junction proteins, which seal the endothelial cells together and produce higher TEER values [36]. Second, the models usually include the presence of a semipermeable basement membrane separating the outer (lumen) and central (brain side) compartments. Lastly, cells, typically astrocytes and or pericytes, are seeded in the central compartment in an effort to mimic the brain microenvironment. The addition of these cells provide cell to cell communication to the endothelial cells in the outer compartment, resulting in the formation of tighter barrier and an increase in TEER [4, 17, 37]. Germane to this work, to re-create the BTB, astrocytes and or pericytes are replaced with tumor cells in the central compartment. In vivo, angiogenesis occurs with the establishment of tumor tissue, resulting in the presence of fenestrations, gaps between the endothelial cells, varied expression of efflux transporters, and an increase in permeability [10, 38].

The use of dyes has been a long-standing method to evaluate the integrity of the BBB and the breakdown of the BTB [39]. Some of the earliest work using dyes dates back to the nineteenth century, where Paul Ehrlich and Edwin Goldmann intravenously injected water-soluble dyes and observed that dyes did not have the ability to freely exchange between the vascular and brain parenchyma compartment (reviewed in [34]). Dyes have also been used as a tool to visualize and qualitatively measure disruption at the BBB [40–44] as well as the BTB [16, 34]. Passive permeability dyes are a simple way to compare rates of diffusion between different models in vivo and in vitro.

In measuring the unrestricted diffusion (the absence of cells) of molecules from the outer chamber to the center chamber, we observed that the diffusion rates (k_in_), from the outer compartment to the central compartment, of all three sized molecules were not significantly different from each other. This data is consistent with previous work showing that if the diameter of each molecule being tested is at least 12× less than the barrier defects, then diffusion will remain constant for all molecules [45].

An interesting aspect of our observations was the similarity of efflux function that existed in the microfluidic model compared to the in vivo BBB [9]. Rhodamine 123 is subject to P-gp mediated efflux at both the BBB and the BTB. When rhodamine 123 and an inhibitor of P-gp are administered concurrently, dye accumulates in brain ~10- to 12-fold higher than in the absence of efflux inhibition [34]. Similarly, in this work, when Verapamil or Cyclosporine A was added to the outer chamber of the microfluidic device, Rhodamine 123 accumulation increased similar to in vivo reports [9]. Further, P-gp function retains function despite barrier breakdown in a number of pathologies [9, 46]. The data herein agree that the degree of efflux function for the BTB, though disrupted, is intact and it retains the ability to restrict drug and dye movement from the vasculature to the brain compartment.

Transwells are a widely used in vitro method to study the BBB. Transwells are cheap, available in high throughput assays, and easy to use. However, there are substantial limitations. First, transport kinetics in transwell systems are strongly influenced by an unstirred water layer that exists on the outer side of the endothelial cells. The unstirred water layer may decrease the apparent permeability rate of lipid soluble and increase to some extent water-soluble molecules. Second, because the cells are grown in a static media, there is no shear stress (or flow) forced on the endothelial cells, which may contribute to the low passive permeability measurements which can be as low as ~74 Ω cm^2^ [47], compared to in vivo values of ~2000 Ω cm^2^ [48]. While a few other in vitro models and microfluidic devices have a flow component [27–31], this microfluidic device is the first commercially available blood-tumor barrier using a microfluidic model seeded with brain-seeking cells and with shear stress similar to that observed in vivo [19] in addition to real-time visualization and quantitation.

## Conclusions

This novel and dynamic microfluidic in vitro BTB model mimics the in vivo barrier with regard to shear stress, permeability, and efflux. Permeability of large molecule dextrans, as well as small molecule dextrans and Rhodamine 123 (with and without inhibitors) were characteristic and relatable to what is seen in vivo. The shear stress from adding flow over HUVECs eliminates the unstirred water layer and allows for different tracers to be added and followed in real time from outer to central compartment. Based on these characteristics, this microfluidic chip shows potential for use in BBB and BTB research. Expanding on these data, future work should entail the use of different drugs, and the comparison of different endothelial cell models to in vivo data with regards to passive permeability and influx/efflux.

## Abbreviations

BBB: blood–brain barrier
P-gp: P-glycoprotein
BTB: blood-tumor barrier
TEER: transendothelial electrical resistance
Free TRD: sulforhodamine 101 Acid Chloride
Rho123: rhodamine 123
TRD 3kDa: Texas Red 3000 MW Dextran
TRD 70 kDa: Texas Red 70,000 MW Dextran
HUVEC: human umbilical vein endothelial cells
EBM-2: endothelial basal medium-2
k_in_: unidirectional uptake transfer constants

## References

[1] Lin NU (2013). CNS metastases in breast cancer: old challenge, new frontiers. Clin Cancer Res.

[2] Daneman R, Prat A (2015). The blood–brain barrier. Cold Spring Harb Perspect Biol.

[3] Serlin Y (2015). Anatomy and physiology of the blood–brain barrier. Semin Cell Dev Biol.

[4] Pardridge WM (2005). Molecular biology of the blood–brain barrier. Mol Biotechnol.

[5] Golden PL, Pardridge WM (1999). P-Glycoprotein on astrocyte foot processes of unfixed isolated human brain capillaries. Brain Res.

[6] Crone C, Christensen O (1981). Electrical resistance of a capillary endothelium. J Gen Physiol.

[7] Olesen SP, Crone C (1983). Electrical resistance of muscle capillary endothelium. Biophys J.

[8] Loscher W, Potschka H (2005). Role of drug efflux transporters in the brain for drug disposition and treatment of brain diseases. Prog Neurobiol.

[9] Adkins CE (2013). P-glycoprotein mediated efflux limits substrate and drug uptake in a preclinical brain metastases of breast cancer model. Front Pharmacol.

[10] Plate KH, Scholz A, Dumont DJ (2012). Tumor angiogenesis and anti-angiogenic therapy in malignant gliomas revisited. Acta Neuropathol.

[11] Folkman J (1971). Tumor angiogenesis: therapeutic implications. N Engl J Med.

[12] Liebner S (2000). Claudin-1 and claudin-5 expression and tight junction morphology are altered in blood vessels of human glioblastoma multiforme. Acta Neuropathol.

[13] Sawada T (1999). Expression of the multidrug-resistance P-glycoprotein (Pgp, MDR-1) by endothelial cells of the neovasculature in central nervous system tumors. Brain Tumor Pathol.

[14] Demeule M (2001). Expression of multidrug-resistance P-glycoprotein (MDR1) in human brain tumors. Int J Cancer.

[15] Lockman PR (2010). Heterogeneous blood-tumor barrier permeability determines drug efficacy in experimental brain metastases of breast cancer. Clin Cancer Res.

[16] Adkins CE (2016). Characterization of passive permeability at the blood-tumor barrier in five preclinical models of brain metastases of breast cancer. Clin Exp Metastasis.

[17] Helms HC (2016). In vitro models of the blood–brain barrier: an overview of commonly used brain endothelial cell culture models and guidelines for their use. J Cereb Blood Flow Metab.

[18] Czupalla CJ, Liebner S, Devraj K (2014). In vitro models of the blood–brain barrier. Methods Mol Biol.

[19] Deosarkar SP (2015). A novel dynamic neonatal blood–brain barrier on a chip. PLoS ONE.

[20] Prabhakarpandian B (2013). SyM-BBB: a microfluidic blood brain barrier model. Lab Chip.

[21] Cucullo L (2011). The role of shear stress in blood–brain barrier endothelial physiology. BMC Neurosci.

[22] Santaguida S (2006). Side by side comparison between dynamic versus static models of blood–brain barrier in vitro: a permeability study. Brain Res.

[23] Loftsson T (2012). Drug permeation through biomembranes: cyclodextrins and the unstirred water layer. Pharmazie.

[24] Korjamo T, Heikkinen AT, Monkkonen J (2009). Analysis of unstirred water layer in in vitro permeability experiments. J Pharm Sci.

[25] Lutgendorf MA (2014). Effect of dexamethasone administered with magnesium sulfate on inflammation-mediated degradation of the blood–brain barrier using an in vitro model. Reprod Sci.

[26] Adriani G (2015). Modeling the blood–brain barrier in a 3D triple co-culture microfluidic system. Conf Proc IEEE Eng Med Biol Soc.

[27] Booth R, Kim H (2012). Characterization of a microfluidic in vitro model of the blood–brain barrier (muBBB). Lab Chip.

[28] Cucullo L (2011). A dynamic in vitro BBB model for the study of immune cell trafficking into the central nervous system. J Cereb Blood Flow Metab.

[29] Herland A (2016). Distinct contributions of astrocytes and pericytes to neuroinflammation identified in a 3D human blood–brain barrier on a chip. PLoS ONE.

[30] Neuhaus W (2006). A novel flow based hollow-fiber blood–brain barrier in vitro model with immortalised cell line PBMEC/C1-2. J Biotechnol.

[31] Griep LM (2013). BBB on chip: microfluidic platform to mechanically and biochemically modulate blood–brain barrier function. Biomed Microdevices.

[32] Cucullo L (2013). A new dynamic in vitro modular capillaries-venules modular system: cerebrovascular physiology in a box. BMC Neurosci.

[33] Koziara JM (2003). In situ blood–brain barrier transport of nanoparticles. Pharm Res.

[34] Mittapalli RK (2013). Quantitative fluorescence microscopy provides high resolution imaging of passive diffusion and P-gp mediated efflux at the in vivo blood–brain barrier. J Neurosci Methods.

[35] Patabendige A, Skinner RA, Abbott NJ (2013). Establishment of a simplified in vitro porcine blood–brain barrier model with high transendothelial electrical resistance. Brain Res.

[36] Zlokovic BV (2008). The blood–brain barrier in health and chronic neurodegenerative disorders. Neuron.

[37] Stanimirovic DB (2015). Blood–brain barrier models: in vitro to in vivo translation in preclinical development of CNS-targeting biotherapeutics. Expert Opin Drug Discov.

[38] Schlageter KE (1999). Microvessel organization and structure in experimental brain tumors: microvessel populations with distinctive structural and functional properties. Microvasc Res.

[39] Hawkins BT, Egleton RD (2006). Fluorescence imaging of blood–brain barrier disruption. J Neurosci Methods.

[40] Bakay L (1956). Ultrasonically produced changes in the blood–brain barrier. AMA Arch Neurol Psychiatry.

[41] Lin SR, Kormano M (1977). Cerebral circulation after cardiac arrest. Microangiographic and protein tracer studies. Stroke.

[42] da Costa JC (1972). Influence of electroconvulsions on the permeability of the blood–brain barrier to trypan blue. Arq Neuropsiquiatr.

[43] Nemeroff CB, Crisley FD (1975). Monosodium L-glutamate-induced convulsions: temporary alteration in blood–brain barrier permeability to plasma proteins. Environ Physiol Biochem.

[44] Schettler T, Shealy CN (1970). Experimental selective alteration of blood–brain barrier by x-irradiation. J Neurosurg.

[45] Mittapalli RK, Adkins CE, Bohn KA, Mohammad AS, Lockman JA, Lockman PR (2017). Quantitative fluorescence microscopy measures vascular pore size in primary and metastatic brain tumors. Cancer Res.

[46] Cordon-Cardo C (1990). Expression of the multidrug resistance gene product (P-glycoprotein) in human normal and tumor tissues. J Histochem Cytochem.

[47] Man S (2008). Human brain microvascular endothelial cells and umbilical vein endothelial cells differentially facilitate leukocyte recruitment and utilize chemokines for T cell migration. Clin Dev Immunol.

[48] Crone C, Olesen SP (1982). Electrical resistance of brain microvascular endothelium. Brain Res.

